# EFFECTIVENESS OF OBESITY INTERVENTION PROGRAMS BASED ON GUIDELINES FOR ADOLESCENT STUDENTS: SYSTEMATIC REVIEW

**DOI:** 10.1590/1984-0462/;2019;37;1;00015

**Published:** 2018-08-09

**Authors:** Vaneza Lira Waldow Wolf, Juan Eduardo Samur-San-Martin, Suzy Ferreira de Sousa, Hemerson Dinis Oliveira Santos, Augusto Gerhart Folmann, Roberto Régis Ribeiro, Gil Guerra-Júnior

**Affiliations:** a Universidade Estadual de Campinas, Campinas, SP, Brasil.; b Universidade Estadual do Oeste do Paraná, Foz de Iguaçu, PR, Brasil.; c Centro Universitário Faculdade Assis Gurgacz, Cascavel, PR, Brasil.

**Keywords:** Child health services, School health, Health behavior, Eating habits, Physical activity., Serviços de saúde da criança, Saúde escolar, Comportamento de saúde, Hábitos alimentares, Atividade física

## Abstract

**Objective:**

To verify the effectiveness of educational interventions based on guidance on physical activity and nutrition in schoolchildren.

**Data sources:**

A systematic search was carried out in four electronic databases containing articles published between October 2007 and January 2017 and addressing educational interventions with emphasis on both nutritional education and physical activity in schoolchildren and adolescents aged 10 to 19 years.

**Data synthesis:**

Twelve articles were selected for this review, of which four included only educational interventions; four made and association between educational interventions, inclusion of healthy foods and physical activity; two made a relation between guidelines and physical activity; and finally, two associated guidelines with consumption of healthy foods.

**Conclusions:**

Interventions based on physical activity and/or nutrition counseling were efficient and showed superior results in studies that associated the practice of physical activity with counseling. However, the need for new studies on educational interventions among schoolchildren and adolescents was made evident.

## RESUMO

**Objetivo:**

Verificar a efetividade de intervenções educacionais baseadas na orientação sobre atividade física e nutrição em escolares.

**Fonte de dados:**

Foi realizada busca sistemática em quatro bancos e bases de dados eletrônicos, com artigos publicados entre outubro de 2007 e janeiro de 2017 que abordaram a realização de intervenções educacionais com ênfase em educação nutricional e atividade física ou exercício físico em escolares com idades entre 10 e 19 anos.

**Síntese dos dados:**

Foram selecionados 12 artigos para esta revisão. Desses estudos, quatro incluíram a intervenção educacional; quatro associaram intervenções educacionais à inclusão de alimentos saudáveis e atividade física; dois estudos relacionaram as orientações e a atividade física; e, por fim, dois ligaram as orientações com o consumo de alimentos saudáveis.

**Conclusões:**

As intervenções baseadas na orientação de atividade física e/ou nutrição foram eficientes, com resultados superiores nos estudos que associaram a prática de atividade física à orientação. Entretanto, observou-se a necessidade da realização de novos estudos que abordem as intervenções educacionais em escolares.

## INTRODUCTION

Childhood obesity has become a major public health problem because of its alarming progress.^1^ ^,^ ^2^ ^,^ ^3^ ^,^ ^4^ According to the World Health Organization (WHO), Latin America and the Caribbean have made progress in preventing and controlling nutritional deficiencies, but one can already identify a rapid increase in rates of overweight and obesity.^2^

This could stem from modernization and urbanization, factors that contributed to a negative change in people’s living habits, as they became more exposed to a variety and diversity of ultra-processed foods, while the consumption of fruit and vegetables decreased. In addition, energy expenditure in work activities dropped, while the access to public transportation improved, and leisure activities became all about games and electronic media.^5^ ^,^ ^6^

Studies have shown^7^ ^,^ ^8^ that the practice of 60 minutes of physical activity per day, that is, any movement that increases energy consumption,^9^ is positively associated with a series of physiological and psychological results, including cardiorespiratory fitness, reduced risk of metabolic diseases, and improvement in body composition profile.

About 50% of physical activities are performed in school environment, since it is an environment suitable to promote activities, either during physical education classes through sports, or with play activities during classes or the break.^10^ ^,^ ^11^ ^,^ ^12^ However, some studies have reported that physical activity classes are inefficient, for exercises are short-lived and have low or very low intensity, which cripples satisfactory benefits.^13^ ^,^ ^14^ ^,^ ^15^

In order to reverse this scenario, programs to stimulate physical activity, adequate nutrient intake and behavioral changes have been developed, as these are essential for health promotion and the prevention of chronic noncommunicable diseases; but one must understand the effects of different types of interventions and their efficacy.^16^ ^,^ ^17^

Thus, the purpose of this investigation was to check the efficacy of educational interventions based on orientation about physical activities and nutrition for adolescent students.

## METHOD

The search process in this review was based on the Preferred Reporting Items for Systematic Reviews and Meta Analyzes (PRISMA),^18^ considering whether intervention studies published between October 2007 and January 2017 were eligible. Searches for keywords of this article were independently identified and selected by two reviewers.

The databases used were portals MEDLINE/PubMed, Latin American and Caribbean Center on Health Sciences Information (BIREME), SPORTDiscus and Embase. The keywords used in search were selected from DeCS (Descriptors in Health Sciences) and Medical Subject Headings (Mesh): “child”, “adolescents”, “education”, “school health services”, “diet”, “exercise” e “clinical trial”. Boolean descriptors “AND” and “OR” were also applied. Keywords and Boolean descriptors were identically organized for search across all databases and portals.

For selection of studies that were relevant for the research objectives and to guarantee homogeneity, the following inclusion criteria were relied on:

1. Studies written in Portuguese and English;
2. Intervention studies carried out at schools with adolescents aged between 10 and 19 years;
3. Studies mentioning educational intervention programs with emphasis to nutritional and/or physical education based on anthropometric profile and body composition;
4. Studies conducted with healthy and/or overweight/obese adolescents.

Studies that described interventions with physical and nutritional activity not associated with educational interventions, not carried out at schools, with students who did not fit the stipulated age range, with specific samples of children with any disease or associating intervention with medicines were not included in the sample.

## RESULTS

Initially, 686 articles were identified in searches on electronic databases. Subsequently, the search was refined with filters in order to exclude literature review articles, resulting in 311 studies.

**Figure 1:**
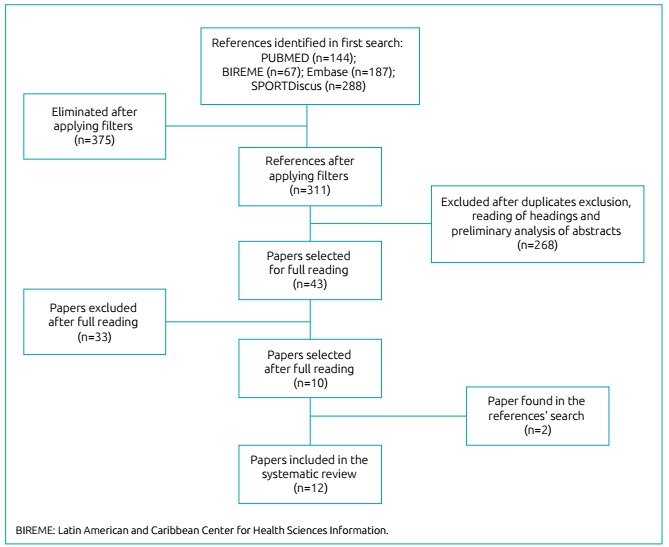
Article selection flow chart.

Subsequently, 43 out of 311 articles were identified and classified as potentially relevant to the study, and therefore read in full. After reading and reviewing them, 31 articles were excluded for not matching our inclusion criteria. Finally, 12 articles were selected (27.9% of the total identified as potential matches). The flowchart of article selection is shown in Figure 1.

### Sample characteristics

Twelve studies were selected, all of them conducted in school environment with adolescents and using methods of educational intervention associated or not with the practice of physical activities and nutritional support. Four studies included only educational intervention,^19^ ^,^ ^20^ ^,^ ^21^ ^,^ ^22^ four made an association between educational interventions, inclusion of healthy foods and physical activity,^23^ ^,^ ^24^ ^,^ ^25^ ^,^ ^26^ two connected guidelines with physical exercise,^27^ ^,^ ^28^ and two made a relation between guidelines and healthy food consumption.^29^ ^,^ ^30^

Four studies^21^ ^,^ ^23^ ^,^ ^24^ ^,^ ^26^ used as inclusion criteria the percentile of body mass index (BMI) greater than 85 or 95%. In Convelli’s study,^27^ subjects selected were supposed to be African-American and be able to read and write in English. On the other hand, studies by Russel et al.^31^ and Willi et al.^30^ used as inclusion criterion the acceptance by schools to reduce prices or the offer of free lunch for at least 50% of selected students and/or ethnic minority groups, in addition to the dropout rate of less than 25%.

Nine^19^ ^,^ ^21^ ^,^ ^23^ ^,^ ^24^ ^,^ ^26^ ^,^ ^27^ ^,^ ^28^ ^,^ ^29^ ^,^ ^30^ out of 12 studies were predominantly developed in the United States and only three in Europe and United Kingdom (Norway, England and the Netherlands). In the study by Fairclough et al.,^22^ 95% of students were British. In the study by Johnston et al.,^23^ ^,^ ^26^ Latin American students were majority.

### Intervention methods

All studies selected covered guidelines based on physical activity or exercise, nutrition, and a healthy lifestyle. Three of them^19^ ^,^ ^20^ ^,^ ^22^ used only methods of counseling as a means of intervention (Table 1).

**Table 1:** Description of studies based on educational interventions only.

Study	Sample	Goals	Intervention	Evaluation	Results
Casazza and Ciccazzo, 2007^19^	3 schools Age range: 13-18 years. CON: n=1,599; CBI: n=2,565; TDI: n=2,573	To compare the results of two methods of service delivery to health education programs in order to determine whether the strategy was effective to adolescents adopting a healthier lifestyle.	11 weeks. CON: recruitment and evaluations. CBI: CD-ROM education, study guide. TDI: lectures, leaflets	Weight; BMI; 24-hour reminder; FFQ; nutritional knowledge questionnaire; PAQ-a; trust on diet and exercise survey; social support survey; questionnaires after the intervention.	BMI: no difference between groups, but TDI group presented pre<post reduction (23.1±0.7 to 22.9±0.7, p<0.001). Knowledge about nutrition: CON = CBI = TDI; CBI< knowledge of nutrition, pre- and post- (40.4+1.25 to 53.2+1.19, p<0.001); TDI> knowledge about pre- and post-nutrition (42.8±1.19 to 50.9±1.12, p=0.003). Physical activity: CON = CBI = TDI. Physical activity pre<post: CBI (17.6±5.92 to 19.6±5.82, p=0.005); Calories intake: pre<post: CBI (p=0.006); TDI (p=0.009). Fat intake: CBI pre<post (p<0.001); CON = TDI. Meals ignored: CBI pre<post (p=0.001). Social support in diet: CBI and TDI pre<post (p<0.001).
Fairclough et al., 2013^22^	12 schools Age range: 10-11 years; CG: n=152; IG=166	To evaluate the effectiveness of the program “Change” in body composition, physical activity practice and food consumption.	20-week intervention and 10-week *follow-up*. IG: 20 weekly lesson plans, homework, spreadsheets and a CD-ROM. CG: received information during school classes, but not related to the program.	Weight, BMI, waist circumference, accelerometers, 24-hour record, APHV and 20m SRT	waist circumference: IG≠CG pre-post: IG> WC (β=-1.63 (95%CI -2.20--1.07) cm, p<0.001). zBMI and LPA: IG≠CG pre-*follow-up*: IG>zBMI (β=-0.24 (95%CI -0.48--0.003), p=0.04); LPA (β=25.97 (95%CI 8.04-43.89) min, p=0.01). WC: OW≠NW, OW>WC (β=-2.82 (95%CI -4.06--1.58) cm, p<0.001) NW (β=-1.34 (95%CI -2.00--0.72) cm, p<0.001). Girls >BMI e zBMI: BMI (β=-0.39 (95%CI -0.81-0.03) kg/m^2^, p=0.07) zBMI (β=-0.18 (95%CI -0.42-0.06) cm, p=0.14). Boys <BMI e zBMI: BMI (β=0.47 (95%CI 0.03-0.91)) kg/m^2^, p=0.04) zIMC (β=0.27 (95%CI 0.02-0.52) cm, p=0.04).
Ezendam et al., 2012^20^	20 schools Age range: 12-13 years; IG: 11 schools (n=485); CG: 9 schools (n=398)	To check the relevance and understanding, as well as news about information provided by FATaintPHAT, and the association between behaviors after four months of follow-up.	10 weeks IG: eight 15-minute lessons via internet. CG: recruitment and assessment	Weight, waist circumference, BMI. Pedometers in five students per class for seven consecutive days, questionnaire after each module, food frequency questionnaire, food frequency questionnaire for snacks, leisure physical activity questionnaire, active transport and screen time, questionnaire for frequency of activities for more than 60 minutes, demographic data questionnaire.	4 months IG≠CG IG> intake of more than 400 mL of sugary beverages. IG> intake of snacks per day (β=0.81 snacks/day). IG< intake of vegetables (β=19.3 g/d). IG< intake of fruit (β=19.3 g/d). IG> steps (β=-10.856 steps/week). No effect on weight, BMI, waist circumference and physical activity. Also no difference in 2-year follow-up.

CON: control; CBI: computer based education; TDI: traditional education; CG: control group; IG: intervention group; BMI: body mass index; FFQ: Food Frequency Questionnaire; PAQ-a: Physical Activity Questionnaire for Adolescents; APHV: age at peak height velocity; 95%CI: 95% confidence interval; zBMI: BMI Z-score; LPA: leisure physical activity; p: statistical significance; WC: waist circumference; OW: overweight or obese; NW: normal weight; :β: regression coefficient; 20m SRT: 20-m shuttle test.

Casazza and Ciccazzo^19^ carried out an 11-month intervention, establishing three groups for the study: the computer-based intervention group (CBI), which received guidelines through a CD ROM developed by nutrition educators and education specialists, each session with about 45 minutes; the second group, “traditional-education intervention” (TDI), which received information through leaflets and 45-minute lectures based on the content in the CD ROM; and the third and last group, “control group” (CON), received no intervention, only follow-up study and evaluations.

The study by Fairclough et al.^22^ was a 20-week intervention with the use of spreadsheets, homework and a CD ROM, plus 20 weekly lessons of 60 minutes each on healthy eating, physical activity and healthy habits for the “Change Group”. The “Control Group” (CG) only received information in school classes, but was not involved in the program or anything specific about diet, health or lifestyle.

Ezembam et al.^20^ also provided information about diet, physical activity, life habits and behavior for the intervention group (IG), but this information was made available through the internet and implemented in school by previously trained teachers. The content was divided into eight modules, each lasting 15 minutes. CG received no intervention.

Three other studies^21^ ^,^ ^29^ ^,^ ^30^ made educational interventions associated with nutritional counselings (Table 2). Kong et al.^21^ conducted an intervention program during the academic year at two school-based health centers (SBHCs) and structured two groups for comparison: intervention group and standard care group. Both groups had an initial visit, in which a summary of all assessments was provided, along with recommendations from the American Academy of Pediatrics. In the Action group, a visit was made every two or three weeks to discuss topics that students would like to bring about, totaling eight visits in the whole academic year. On the first visit, participants received a DVD, a DVD player and leaflets. Caregivers were given leaflets on strategies to reduce the risk of obesity.

**Table 2:** Description of studies based on educational interventions associated with nutritional counseling.

Study	Sample	Goals	Intervention	Evaluation	Results
Kong et al., 2013^21^	Two schools Students and parents/caregivers Action: n=28 Mean age: 15±1 year. SCG: n=23 Mean age:14.6±0.7	To evaluate whether the Action program could reduce zBMI in IG when compared to SCG.	During school year. Action: eight motivational meetings, DVD, assessment results, print material, recommendations by the American Academy of Pediatrics + information leaflets for parents. SCG: one visit upon intervention start, brochure on “balance for a healthy life”, assessment results, recommendations by the American Academy of Pediatrics.	Weight, BMI, hematocrit in girls, cholesterol, FitnessGram Pacer, research on changes in living habits, diabetes and liver fat tests in obese or overweight students with family history of diabetes.	zBMI pre-post: Action ≠SCG, Action>zBMI =(=-0.3 (95%CI -0.6-0.3), SCG (=0.2 (95%CI -0.1-0.8) (p=0.04). WC pre-post: Action ≠ SCG, Action >WC =(=-0.0 (95%CI -1.4-1.4), SCG (=1.7 (95%CI 0.4-2.9) (p=0.04). Screen time on week days pre-post: Action ≠SCG: Action>SCG =(=-0.4 (95%CI -1.0-0.2), SCG (= 0.2 (95%CI 0.3-0.6) (p=0.03).
Jago et al., 2011^29^	42 schools Mean age: 11.3±0.6 IG: n=3,222; CG: n=3,191.	To investigate whether the intervention program “Healthy” led participants to increase their levels of physical activity, and the prevalence of metabolic diseases, comparing IG with CG.	2,5 years. IG: inclusion of healthy food in diet, Flash, social marketing campaign and more active physical education classes social. CG: recruitment and assessment.	Weight, BMI, waist circumference, 20-m shuttle test (20-MST), SAPAC, MPVA time, lab tests (LDL, HDL cholesterol) and blood pressure.	No statistically significant difference in any test.
Willi et al., 2012^30^	42 schools Age range: 11-14 years IG: n=2,185; CG: n=2,178	To examine the effects of the intervention program “Healthy” on cardiovascular risk factors.	2.5 years. IG: changes in eating habits and in physical activity practice, social marketing campaign to reinforce messages and images. CG: recruitment and assessment.	Weight, BMI, lab tests (LDL, HDL cholesterol) and blood pressure.	No statistically significant difference in any test.

SCG: standard care group; CG: control group; IG: intervention group; BMI: body mass index; zBMI: BMI Z-score; Action: intervention group; Flash: Fun Learning Activities for Student Health; SAPAC: Self-Administered Physical Activity Checklist; MPVA: moderate to vigorous physical activity; LDL: low density lipoprotein; HDL: high density lipoprotein; : mean; 95%CI: 95% confidence interval; WC: waist circumference; p: statistical significance.

Jago et al.^29^ and Willi et al.^30^ developed a two-and-a-half-year intervention program aimed at changing eating and physical activity habits in school environment, using social marketing as a complement for intervention. Jago et al.,^29^ unlike Willi et al.,^30^ reported offering five Fun Learning Activities for Student Health (Flash) modules and five social marketing themes based on self-knowledge, understanding, decision-making, physical activity habits, sedentary time, energy balance and life choices. The last component was to increase the time spent in physical activities, which was not mentioned in the study by Willi et al.^30^ None of the studies^29^ ^,^ ^30^ applied interventional techniques to CG.

Three other studies^23^ ^,^ ^24^ ^,^ ^26^ carried out educational interventions associated with physical activity orientation or practice (Table 3). Love-Osborne et al.^24^ conducted preventive services and evaluations in two groups: IG and CG. The IG was oriented to self-monitor weight and lifestyles on a weekly basis and received nutritional and physical activity guidelines according to the students’ goals. They were also able to choose to return in different periods (two weeks, one or two months) and were referred to resources for physical activity and healthy eating at the school or in the community. In addition, IG was allocated in two subgroups: one receiving two weekly text messages (TM) reinforcing the guidelines and reminding them of self-monitoring; the other grupo did not receive messages (NTMs).

**Table 3:** Description of studies based on educational interventions associated with physical activity orientation or practice.

Study	Sample	Goals	Intervention	Evaluation	Results
Love-Osborne et al., 2014^24^	Two schools CG: n=83 Mean age: 16±1.5 years. IG: n=82 Mean age: 15.7±1.5 years.	To evaluate whether the program “HE” promotes more contact with students and improves lifestyle and results in BMI in overweight adolescents.	IG: healthy eating in school environment or the community, orientation for 1 hour of daily physical activity, self-monitoring by weekly records; IG TM: received electronic messages; IG NMT: did not receive messages. CG: preventive service, assessments	Peso, IMC, hematocrit in girls, cholesterol, FitnessGram Pacer, research on changes in living habits, diabetes and liver fat tests in obese or overweight students with family history of diabetes.	zBMI: 55% of IG and 72% of CG had zIMC reduced or stable (p=0.025); 40% of CG and 18% of IG had zIMC reduced (p=0.02). Participation in sports: 47% of CG and 28% of IG (p=0.02) Sports practice: CG>IG (47% *versus* 28% in IG; p=0.02) Students aged > 15 years had better results in zIMC reduction (p=0.03).
Johnston et al., 2007^23^	71 schoolchildren. Age range: 10-14 years. II: n=46 SH: n=25	To evaluate the effect of a randomized intervention in school environment as to weight loss in overweight schoolchildren.	24 weeks II: 12 weeks of physical activity, nutritional counseling, inclusion of healthy foods, monthly meetings with parents, guidance material and 12 biweekly follow-up sessions. SH: book *“Trim Kids”* for both students and parents.	Weight, BMI, lab tests (cholesterol, triglycerides, HDL, LDL, glucose), blood pressure and body fat percentage with electric bio-impedance scale.	IMC: II≠SH, II>IMC (p=0.004). II>zBMI at three and six months (p=0.001). Body fat percentage: II≠SH at six months, II> body fat percentage (p=0.001)
Johnston et al., 2010^26^	71 schoolchildren. Age range: 10-14 years. II: n=40 SH: n=20	To verify long-term effectiveness of the intervention program carried out by Johnston *et al.* ^23^.	1 and 2-year follow-up of the study conducted by Johnston et al.^23^.	Weight, BMI, lab tests (cholesterol, triglycerides, HDL, LDL, glucose), blood pressure and body fat percentage with electric bio-impedance scale and triceps fold.	1 and 2-year follow-up II≠ SH II >zBMI, SH (p<0.001); II: 1 and 2 years (p<0.001, p<0.05). 1-year *follow-up.* II>BMI (p<0.001); II>Weight (p<0.001) II>overweight percentage (p<0.001). II>cholesterol (p<0.05), II>triglycerides (p<0.05) II>triceps fold (p<0.01).

CG: control group; IG: intervention group; II: intensive intervention; SH: self-help only condition; HE: Health Educator; BMI: body mass index; TM: text messages; NTM: no text messages; HDL: high density lipoprotein; LDL: low density lipoprotein; zBMI: BMI Z score; p: statistical significance. : mean

The intervention by Johnston et al.^23^ ^,^ ^26^ lasted 24 weeks. The “intensive intervention” (II) group consisted of daily sessions in the first 12 weeks: four days in the week of exercise or physical activity for 30 to 35 minutes with intensity equivalent to 60-85% of maximum heart rate (HR_max_), and one day a week for nutritional advice. Healthy food options were also offered as snacks and breakfast, as well as support materials for students and their relatives, plus a monthly meeting with them. In the subsequent 12 weeks, the sessions were biweekly. Students and their parents in self-help only condition (SH) were instructed to use the book “Trim Kids”^32^ and took part in 12 weekly sessions, followed by maintenance activities.

Three other studies^25^ ^,^ ^27^ ^,^ ^28^ addressed educational interventions associated with the practice of physical activity or exercise and nutritional support (Table 4). Like Johnston et al.,^23^ two other studies^27^ ^,^ ^28^ used HR_max_ as a marker of exercise intensity.

**Table 4:** Description of studies based on educational interventions associated with physical activity orientation or practice and nutritional counseling.

Study	Sample	Goals	Intervention	Evaluation	Results
McFarlin et al., 2013^28^	Age range: 12-14 years IG: n=152; SH: n=69	To validate a school-based intervention along 12 months, using plasma, resistin, adipokine, leptin concentrations and biological results.	IG: physical activity practice, nutritional counseling, SH: instruction manual on weight loss and weight maintenance.	Weight, BMI, blood plasma (resistin, adiponectin and leptin).	Pre-post zBMI: IG ≠SH 6 months, IG>zBMI IG (-0.211±0.005) *versus* SH (-0.173±0.004) (p<0.05); 12 months IG (-0.105±0.004) *versus* SH (-0.068±0.006). Pre-post six months resistin = IG≠ SH, IG> resistin in six months (p=0.001). Adipokine= IG ≠SH, six months (p<0.001); 12 months (p<0.001). Pre-post 12 months Leptin: IG≠SH, IG>leptin (p=0.013)
Covell, 2008^27^	62 schoolchildren Age range: 14-17 years IG: n=37 CG: n=25	To raise the level of knowledge about health, increase daily exercise practice, increase intake of fruits and vegetables, maintain blood pressure levels.	Nine weeks. IG: classes with lectures and discussions, physical exercise, guidelines on diet and exercise and self-reported food consumption and weekly practice of exercises. CG: Life Management Class, theoretical intervention.	Blood pressure, demographic data, physical activity questionnaire, nutrition questionnaire with 2-day recall report and test for knowledge about health.	Pre-post knowledge: IG≠CG, IG<knowledge (p=0.0001). Pre-post fruit and vegetable intake IG≠CG, IG< fruit and vegetable intake (p=0.0001). Pre-post physical activity practice: IG≠CG, IG> physical activity practice (p=0.0001), superior among girls.
Grydeland et al., 2014^25^	37 schools. Mean age: 11,2 ± 0,3 IG: n=465 CG: n=859	To investigate the effectiveness of a 20-month HEIA intervention program.	IG: classes, posters, inclusion of fruits and vegetables in diet, regular physical activity, campaigns for active transportation, online counseling, school and parents meetings, information leaflets for parents and a machine for cutting and selling fruit and vegetables. CG: recruitment and assessment.	Weight, BMI, height-to-waist ratio, pubertal stage, parents’ educational level, demographic characteristics.	No significant differences in body composition between groups. Parents with low educational level, pre-post: IG >CG: 0.426 (0.422 to 0.430) CG 0.420 (0.417 to 0.423) (p=0.020) Parents with high educational level: IG>CG, pre - = 18.6 (95%CI 18.5-18.8) -; post- =18.4 (95%CI 18.2-18.6) (p=0.027) Girls IG≠CG pre-post IG>BMI, IG - =19.2 (95%CI 19.1-19.3); CG - =19.0 (95%CI 18.8-19.3) (p=0.02); IG>zBMI, IG - =0.03 (95%CI -0.01-0.08); CG - =-0.8 (95%CI -0.14--0.02) (p=0.003).

IG: intervention group; SH: self-help only condition; CG: control group; BMI: body mass index; zBMI: BMI Z score; p: statistical significance; : mean; 95%CI: 95% confidence interval.

A study by McFarlin et al.^28^ lasted 12 months. The first three months were intended for physical exercise four days a week with intensity of 60 to 85% of predicted HR_max_ and one day of nutritional counseling. The three months that followed were allocated to weekly sessions. The program was followed for another six months, but without assistance. The SH group was handed an instruction manual about weight loss and maintenance but received no formal guidance.

Covelli^27^ conducted a nine-week intervention program with two groups. The IG consisted of two 90-minute lessons per week - one day for lectures and discussions, so as to promote a cognitive behavioral change in health knowledge, concepts about health, nutrition and exercise; and a day focused on physical exercises: 30 minutes of exercise monitored as to target heart rate and duration. Students were also encouraged to include fruits and vegetables in their diet, reduce sodium intake, practice at least 30 minutes of physical activity per day and self-report food consumption and exercise or physical activity practiced along the week. The CG attended the theoretical intervention Life Management Class throughout the program.

The last intervention technique found in studies was a 20-month program conducted by Grydeland et al.,^25^ in which participants attended classes, were exposed to posters, were oriented as to inclusion of fruits and vegetables, regular physical activity of 10 minutes during regular classes, campaigns for active transportation and online counseling on physical activity, screen time, and nutrition. In spare time, group meetings were held in school environment and with the presence of parents, where they also received information leaflets. In school environment, a machine was made available for cutting and selling fruits and vegetables, making it easier to consume these foods. In addition, all professionals involved in the research and intervention took part in meetings aimed at getting new material and attended courses of inspiration for the program.

### Assessment and results

### Anthropometric measurements and waist circumference

Most studies^19^ ^,^ ^20^ ^,^ ^21^ ^,^ ^22^ ^,^ ^23^ ^,^ ^24^ ^,^ ^25^ ^,^ ^26^ ^,^ ^27^ ^,^ ^28^ ^,^ ^29^ used weight and BMI measurement as a parameter for evaluation. Out of these, six^21^ ^,^ ^22^ ^,^ ^23^ ^,^ ^25^ ^,^ ^27^ ^,^ ^28^ reported significant differences in BMI reduction in the groups that received the intervention. One of them reported a relevant result as to BMI reduction in CG.^24^ The study conducted by Casazza and Ciccazzo^19^ pointed out a decrease in BMI, but no significant difference after 11 weeks of intervention. The other studies^19^ ^,^ ^20^ ^,^ ^29^ ^,^ ^30^ did not found significant differences between groups after intervention.

Two studies^22^ ^,^ ^25^ also showed that girls had better BMI scores when compared to boys after intervention. Upon body weight analysis, only one study^27^ reported a significant difference after one year of follow-up.

In addition to body weight and BMI, waist circumference was also assessed in four studies.^20^ ^,^ ^22^ ^,^ ^29^ In two of them,^21^ ^,^ ^22^ significant differences were pointed out in waist circumference reduction after the intervention period. Fairclough et al.^22^ concluded that overweight or obese students had more significant results in reducing waist circumference than normal-weight students. Only one study^26^ measured triceps folds and reported statistically positive results in the group receiving the intervention. Another factor evaluated in only two studies^23^ ^,^ ^26^ was body composition, with significant reduction in fat percentage upon assessment after intervention, but no differences were seen in the follow-up analysis.^26^

#### Physical activity, nutrition and knowledge about health 

Physical activity and food intake were assessed in three studies.^19^ ^,^ ^20^ ^,^ ^21^ ^,^ ^27^ Only one^27^ found a significant difference between CG and IG as to physical activity, and two studies^19^ ^,^ ^21^ did not find a significant difference between groups. However, Casazza and Ciccazzo^19^ noticed an increase in the practice of physical activity in the group that was handed the educational CD-ROM and a study guide. The same authors also noted significant differences as to reduction of calorie consumption and increase of social support to diet in the TDI and CBI groups, and also a reduction in fat consumption and in number of meals ignored in CBI.

Another study that showed improvement in nutritional profile and practice of physical activity after the intervention was that of Ezendam et al.,^20^ who reported increase in number of steps, reduction in consumption of sugary drinks with more than 400 mL, decrease in consumption of snacks and increased intake of fruits and vegetables. Kong et al.^21^ reported a reduction in screen time in the group that received intervention.

Contrary to other studies, Love-Osborne et al.^24^ mentioned a significant difference in sports practice, but it was higher in CG compared to GI.

#### Biochemical markers

Among studies included in this systematic review, seven conducted laboratory tests.^21^ ^,^ ^23^ ^,^ ^24^ ^,^ ^26^ ^,^ ^28^ ^,^ ^29^ ^,^ ^30^ Of these, only two reported significant alteration after intervention: Mcfarlin et al.^28^ pointed an increase in resistin in IG after six months, increase in leptin after 12 months and improvement in adipocytokine levels after 6 and 12 months of intervention; and Johnston et al.^26^ reported a reduction in cholesterol and triglycerides levels in IG.

#### Blood pressure

Blood pressure was measured in five studies,^16^ ^,^ ^19^ ^,^ ^20^ ^,^ ^22^ ^,^ ^23^ but none reported significant differences after intervention.

## DISCUSSION

This review included 12 intervention studies that covered programs with educational guidelines on physical activity and/or nutrition concepts, associated or not with practice of physical activity and dietary modifications. Of these, 83% reported significant results in parameters evaluated after interventions.

Oosterhoff^33^ and Waters,^34^ after conducting a systematic review, found significant results BMI reduction associated with incentive to physical activity practice, and food quality improvement. They also stated that such interventions can help maintain body weight and behavioral change. These studies corroborate the finding of ours, in which the interventions were found to succeed in causing positive changes in life habits by increasing consumption of fruits and vegetables, as well as the levels of physical activity or exercises,^19^ ^,^ ^22^ ^,^ ^24^ ^,^ ^27^ reducing the number of meals ignored, intake of calories, fats, snacks and sugary drinks,^19^ ^,^ ^20^ ^,^ ^27^ and also screen time.^21^ To obtain such result, studies used different methods of intervention. For the variable BMI, those using physical activity or exercise as an intervention method were efficient. However, for the other variables, it was not possible to identify which techniques had a better effect, since the evaluation methods were different. However, studies that dealt with a similar theme reported that results are inconsistent, short-term and do not necessarily mean a reduction in health risk factors.^33^ ^,^ ^34^ ^,^ ^35^

The other studies that analyzed the effects of physical activity on adolescents reported effective results for the improvement of health conditions;^36^ ^,^ ^37^ however, the need to determine the quality of physical education classes, stimulating the practice of pleasant and fun physical activities, without competitive or performance-based guidance, is brought about.^38^ Healthy lifestyle habits, such as physical activity practice and quality food consumption are also encouraged through educational support, so that behavioral changes are sustainable and related to the construction of intrinsic motivation to maintain the desired behavior until adulthood.^33^ ^,^ ^35^ In addition, the studies carried out over the past ten years to date have highlighted the need to develop effective strategies for interventions, involving a more active participation of teachers and parents, inclusion of educational components with aided by technology, as well as social and environmental factors in physical activity, stimulation of healthy life habits outside school and longer intervention time.^20^ ^,^ ^21^ ^,^ ^22^ ^,^ ^23^ ^,^ ^24^ ^,^ ^27^ ^,^ ^29^ ^,^ ^33^ ^,^ ^34^ ^,^ ^41^

Among the limitations presented, studies that did not find significant results^29^ ^,^ ^30^ reported that the outcomes achieved during the intervention period were lost in summer vacations, preventing their detection. Results were also imprecise and difficult to prove, since the students had normal baseline values and data obtained from questionnaires and information did not show good reliability.^20^ ^,^ ^30^ Limitations were also observed in school selection and attributed to the difficulty in finding subjects who would agree to participate in interventions, as well as teachers’ reluctance to let students leave classrooms to participate in intervention sessions.^19^ ^,^ ^22^ ^,^ ^24^ Willi et al.^30^ mentioned the students’ lack of involvement in research. This factor may have hampered the effect of interventions, since the participation of teachers and family members is valuable to cause healthy lifestyle changes.^26^

In view of the above, interventions in school environments were efficient in improving health and behavioral condition of adolescents, nevertheless caution is necessary to control possible limitations, considering that the school period is essential for acquiring knowledge, modifying life habits or creating healthier ones.

We can conclude that interventions for physical activity and/or nutrition counseling were efficient, but the studies that associated physical activity with interventions had better and more significant results when compared to those that made use of programs based on orientation only.
